# Preclinical evaluation of multivalent vaccine combinations against *Shigella* and *Salmonella* infections

**DOI:** 10.3389/fimmu.2026.1807547

**Published:** 2026-04-22

**Authors:** Roberta Di Benedetto, Rebecca Nappini, Salvatore Gemmellaro, Federica Boretto, Valentina Caradonna, Martina Carducci, Paola Cescutti, Omar Rossi, Francesca Mancini, Francesco Berlanda Scorza, Simona Rondini, Carlo Giannelli, Renzo Alfini, Francesca Micoli

**Affiliations:** 1 GSK Vaccines Institute for Global Health (GVGH), Siena, Italy; 2 Department of Life Sciences, University of Trieste, Trieste, Italy

**Keywords:** antimicrobial resistance, glycoconjugates, GMMA, multi-component vaccines, *Salmonella*, *Shigella*

## Abstract

Shigellosis is an intestinal infection causing severe and often life-threatening diarrheal disease, with high prevalence in children under five years of age in low- and middle-income countries. Given the rise of antimicrobial resistance to *Shigella*, the World Health Organization has included this pathogen among those for which the development of new interventions is a global health priority. No vaccines against *Shigella* are licensed yet, but several candidates based on the O-antigen portion of lipopolysaccharides are in clinical development, and combining a *Shigella* vaccine with another vaccine has recently been recommended to broaden protection while minimizing the need for additional injections in an already crowded childhood immunization schedule. Here, in animal models, we present a novel combination vaccine strategy: a tetravalent GMMA−based *Shigella* vaccine co-formulated with a bivalent *Salmonella* glycoconjugate vaccine targeting *S.* Typhi and *S.* Paratyphi A, the causative agents of typhoid and paratyphoid fever. We demonstrate the technical feasibility of combining all six antigens without major impact on the humoral immune response to any component. Moreover, we show that *Shigella* GMMA can serve as a carrier for the *S.* Paratyphi A O−antigen, enabling a formulation with fewer distinct components. The resulting vaccine candidate, combining *Shigella* and *Salmonella* antigens, has the potential to enhance vaccine acceptance and uptake, facilitate programmatic roll-out, reduce delivery costs, and contribute to reducing disease burden and antimicrobial resistance.

## Introduction

*Shigella* spp. are the causative agents of shigellosis, an intestinal infection that can lead to severe and often life-threatening diarrheal disease (1). *Shigella* is estimated to cause 80–165 million cases worldwide (2) resulting in 148,202 deaths in 2019 (3). The majority of cases and deaths occur among children under five years of age, primarily in low- and middle-income countries (LMICs), with a geographic concentration in sub-Saharan Africa and South Asia (4–6).

The rise of antimicrobial resistance (AMR) to *Shigella* (7–9) makes this disease an even greater global health concern. Indeed, this pathogen has been included by the World Health Organization (WHO) among those for which the development of new interventions is a global health priority (10).

No vaccines are available against *Shigella* to date, but several candidates are in clinical development (11, 12), many of them based on the O-antigen (OAg) portion of lipopolysaccharide (LPS) (13–15), recognized as key target for protective immunity (16, 17). The heterogeneous distribution of *Shigella* serotypes (5, 18–20) constitutes a challenge for the development of an effective vaccine, implying that a multivalent vaccine is required to cover the most epidemiologically relevant serotypes (9). Based on global epidemiological data, the most advanced *Shigella* vaccines share a 4-valent composition consisting of *Shigella flexneri* 2a, 3a, and either 6 or 1b, along with *Shigella sonnei* (20, 21). A 4-valent vaccine is estimated to cover around 70% of *Shigella* cases based on the serotypes included in the vaccine. Additionally, it has the potential for broader coverage, exceeding 80%, due to cross-reactivity associated with common epitopes in *S. flexneri* OAg structures (20, 22).

A clinical development pathway for licensure of a *Shigella* vaccine has been proposed (23), though discussions remain ongoing regarding the precise regulatory pathway and requirements. However, even a highly efficacious vaccine may not be recommended, prioritized, purchased, or widely adopted in LMICs, due to increasingly crowded and costly childhood immunization programs and the recent introduction of other high-priority vaccines (24, 25). For this reason, combination of a *Shigella* vaccine with another vaccine would increase its attractiveness, as recommended by GAVI in the last Vaccine Investment Strategy (VIS) 2024 (26).

Different serovars of *Salmonella enterica* cause systemic diseases in humans. Among these, *Salmonella* Typhi and *Salmonella* Paratyphi A are responsible for typhoid and paratyphoid fever, respectively, and present a high incidence especially in South and Southeast Asia (27, 28). Antibiotics are widely used but increasing levels of AMR are limiting their effectiveness (29, 30). Just like *Shigella*, *Salmonella* serovars are also included in the WHO high priority list of AMR pathogens (10). While several licensed and WHO–prequalified vaccines are available for typhoid fever, no vaccine currently exists against *S.* Paratyphi A. However, bivalent candidates targeting both serovars are in development (29).

Here, for the first time, we have combined a tetravalent Generalized Modules for Membrane Antigens (GMMA)-based *Shigella* vaccine (31) with a bivalent *Salmonella* vaccine, consisting of the licensed typhoid conjugate vaccine (Vi-CRM_197_, TCV) and the *S.* Paratyphi A O:2-CRM_197_ glycoconjugate (32). After promising results in phase 1, the 4-valent *Shigella* GMMA vaccine, named altSonflex1-2-3, targeting *S. sonnei* and *S. flexneri* 1b, 2a and 3a serotypes, is currently being tested in phase 2 clinical trials in 9-months old infants in Kenya, where shigellosis is endemic (NCT05073003 and NCT06663436) (33). On the other hand, the bivalent *Salmonella* conjugate vaccine has recently been tested in a phase 1 clinical trial in European adults (NCT05613205), showing very strong immune response for both components.

We have formulated this novel hexavalent candidate vaccine and tested it in different animal models to evaluate its immunogenicity and if any immuno-interference would occur among the different polysaccharide (PS) components.

In addition, we conjugated the *S.* Paratyphi A OAg (O:2) to *Shigella* GMMA (*S. sonnei* GMMA), to explore the possibility of simplifying the final formulation by leveraging GMMA’s ability to act as a carrier for heterologous PS, which has already been verified in our laboratories with different antigens (34, 35).

A multivalent vaccine tackling *Shigella* and *Salmonella* simultaneously could address a significant unmet need. As these two pathogens coexist in many geographical areas, combining them into a single infant vaccine could substantially contribute to AMR reduction, increase commercial attractiveness through reduced cost of goods, and improve acceptance among end users and health-care providers, ultimately leading to higher and more equitable vaccination coverage.

## Results

### Hexavalent formulation against both *Shigella* and *Salmonella*

The four *Shigella* GMMA from *S. sonnei*, *S. flexneri* 1b, 2a and 3a were combined with *S.* Typhi Vi-CRM_197_ and *S.* Paratyphi A O:2-CRM_197_ glycoconjugates. The different drug substances, whose main characteristics are reported in **Supplementary Table 1**, were combined at their highest envisaged human dose, corresponding to 15 μg of each *Shigella* OAg and 25 µg of each *Salmonella* PS. The 4-valent *Shigella* vaccine uses Alhydrogel as adsorbent (31), while Vi-CRM_197_ is licensed without any adjuvant. To account for both situations, we formulated the vaccine with and without Alhydrogel.

When Alhydrogel was included, formulation conditions were optimized to have GMMA completely adsorbed and Vi-CRM_197_ mainly not adsorbed. By playing with the final concentration of phosphate buffer, it was possible to modulate the adsorption of O:2-CRM_197_. Indeed, GMMA were diluted in saline and Alhydrogel was added. After continuous stirring, sodium phosphate buffer was introduced, followed by addition of the glycoconjugates. The presence of the two *Salmonella* glycoconjugates did not affect the adsorption of GMMA on Alhydrogel, which was >95%, as verified by analyzing lipid A content in the supernatant. By modulating the final phosphate concentration from 5 to 50 mM, the adsorption of O:2-CRM_197_ ranged from 8 to 79%, while Vi-CRM_197_ remained mainly not-adsorbed (>50%). Furthermore, adding O:2-CRM_197_ before or after the phosphate buffer, as well as changing the order of addition of the two conjugates, did not influence their adsorption (**Table 1**). Real-time stability testing on the various formulations is underway to evaluate the impact of antigen adsorption state on long-term stability.

**Table 1:** Different conditions applied for the hexavalent formulation and resulting formulates characterization.

Formulation	Formulation procedure	Not adsorbed O:2 %	Not adsorbed Vi %	Not adsorbed Lipid A %
Hexavalent_ 5 mM NaPi	Condition 1: GMMA adsorption on Alum (2 hours), NaPi and NaCl addition (30 minutes), conjugates addition (15 h ± 4 hours)	<18	na	<1
Hexavalent_ 10 mM NaPi		<18	55	<1
Hexavalent_ 20 mM Napi		<18	65	<1
Hexavalent_ 50 mM NaPi		66	70	<1
Hexavalent_ 20 mM NaPi	Condition 2: GMMA adsorption on Alum (2 hours), NaPi and NaCl addition (30 minutes), Vi-CRM_197_ addition (1 hour), O:2-CRM_197_ addition (15 h ± 4 hours)	<18	71	<1
Hexavalent_ 40 mM NaPi		37	81	<1
Hexavalent_ 50 mM NaPi		79	85	<1
Hexavalent_ 20 mM NaPi	Condition 3: GMMA adsorption on Alum (2 hours), O:2-CRM_197_ addition (1 hour), NaPi and NaCl addition (15 minutes), Vi-CRM_197_ addition (15 h ± 4 hours)	8	77	1

All formulations have the following final composition: 30 μg/mL each *Shigella* OAg; 50 μg/mL Vi; 50 μg/mL O:2; 0.7 mg/mL Al^3+^; 154 mM NaCl. The concentration of sodium phosphate (NaPi) for each formulation is reported in the first column of the Table.

Note that when minor is reported, this means that all replicates were below the lower limit of quantification of the analyses. When formulations were re-prepared and analyzed CV% ≤ 17% were found for the parameters tested.

### Immunogenicity study of the hexavalent formulation in mice

The hexavalent formulations without and with Alhydrogel (with GMMA and O:2-CRM_197_ adsorbed, **Table 1** condition 3) were tested in mice. Animals were injected twice intraperitoneally (i.p.), 28 days apart, at 1/200 of the highest expected human dose. The 4-valent *Shigella* GMMA (31, 36), with Alhydrogel, and the bivalent *Salmonella* glycoconjugates (32) (Alfini R, submitted), with and without Alhydrogel, were used as comparators.

**Figure 1:**
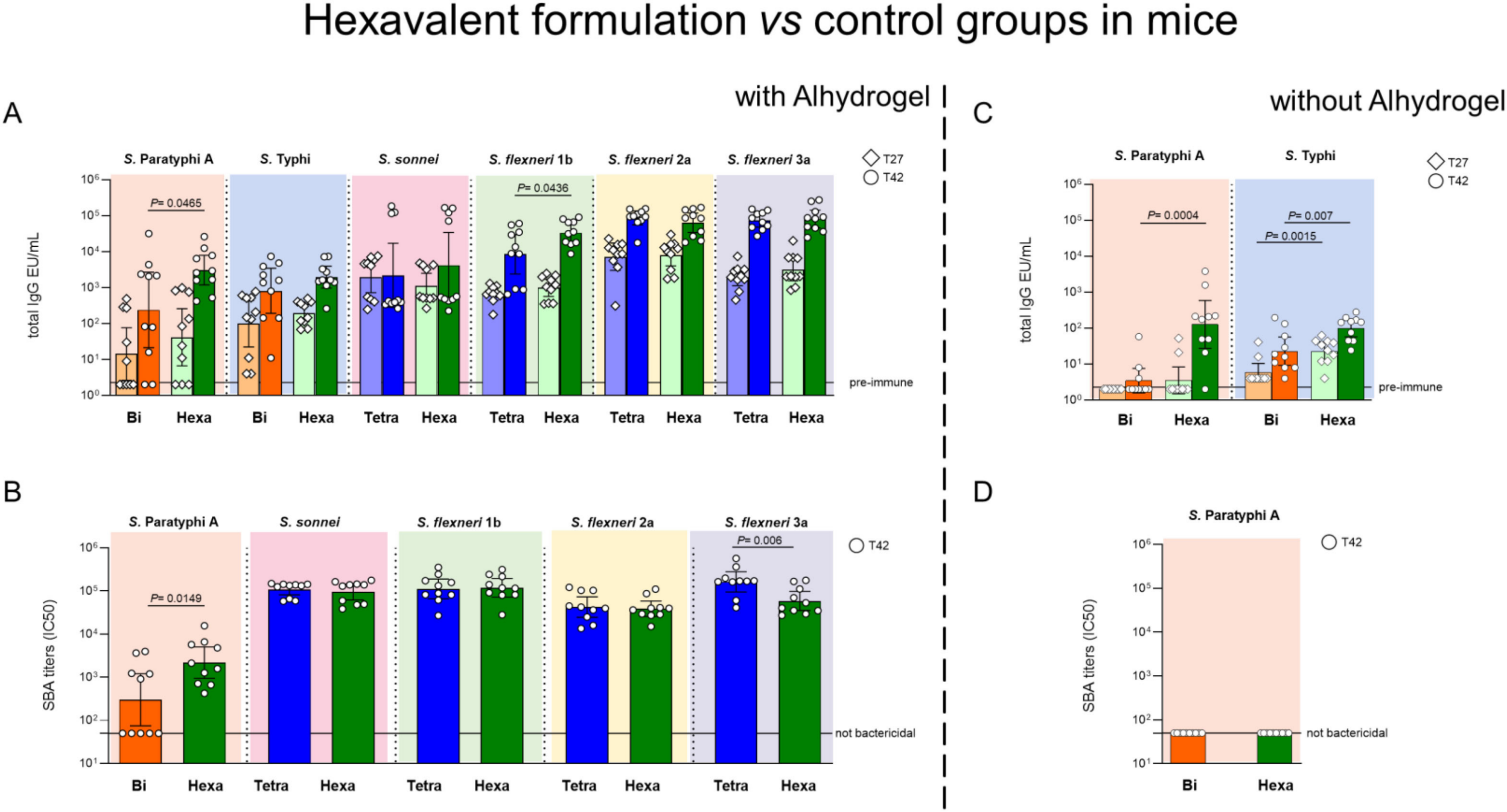
Hexavalent formulation (Hexa, green) tested in mice with and without Alhydrogel in comparison to bivalent *Salmonella* glycoconjugates (Bi, orange) and *Shigella* tetravalent GMMA (Tetra, blue). CD1 mice were immunized intraperitoneally (i.p.) at day 0 and 28 with 75 ng/dose of each *Shigella* GMMA OAg and 125 ng/dose of each *Salmonella* glycoconjugate PS. Concentration of Alhydrogel, if present, was 0.7 mg/mL (Al^3+^). Sera collected at days 27 (T27) and 42 (T42) were analyzed by **(A, C)** ELISA for OAg-specific (*S. sonnei*, *S. flexneri* and *S.* Paratyphi A) or Vi total IgG (EU/mL). Sera collected at T42 were analyzed by **(B, D)** SBA for bactericidal titers expressed as IC50. Summary graphs of geometric mean units (bars) and individual levels (dots) are reported. .

Importantly, when comparing the hexavalent formulation with Alhydrogel to either the corresponding bivalent *Salmonella* or 4-valent *Shigella* formulations, no evidence of negative immuno-interference was observed. Similar IgG responses (after both the first and the second injections) and bactericidal titers (measured after second injection) were induced by all formulations (**Figures 1A, B**) against all components. The only exception was *S. flexneri* 3a, for which the hexavalent formulation induced lower bactericidal titers compared to the 4-valent *Shigella* GMMA (**Figure 1B**). For *Salmonella* Paratyphi A and *S. flexneri* 1b, the hexavalent formulation generated higher responses than the control groups in the enzyme−linked immunosorbent assay (ELISA) and in the serum bactericidal assay (SBA) for *S.* Paratyphi A only (**Figures 1A, B**).

Interestingly, in the absence of Alhydrogel, the presence of GMMA in the hexavalent formulation led to significantly higher anti-Vi (at both timepoints) and anti-O:2 (at day 42) IgG responses, compared to those induced by the bivalent vaccine (**Figure 1C**). However, sera against *S.* Paratyphi A were not bactericidal, even in the presence of GMMA (**Figure 1D**).

Impact of Alhydrogel on IgG responses induced by the hexavalent formulation was also assessed. Anti-Vi, -O:2, and -*Shigella flexneri* 1b and 3a IgG were higher when Alhydrogel was present in the formulation, whereas anti-*S. sonnei* and S. *flexneri* 2a IgG responses were similar with or without it (**Supplementary Figure 1A**). Additionally, the hexavalent formulation with Alhydrogel induced higher bactericidal titers against *S.* Paratyphi A, *Shigella flexneri* 1b, 2a and 3a. No difference was observed for *S. sonnei* (**Supplementary Figure 1B**). To be noted that, in the absence of Alhydrogel, no bactericidal titers were elicited against *S.* Paratyphi A (**Supplementary Figure 1B**).

Only for the O:2 component of the hexavalent formulation in the absence of Alhydrogel, a second injection was needed for eliciting a significant response in mice. After the second injection (T42), the IgG responses increased further for all components, with or without Alhydrogel. Only for *S. sonnei* there was not a statistically significant increase of IgG post second injection for the formulation with Alhydrogel (*P* value > 0.05).

### Immunogenicity study of the hexavalent formulation in rats

The hexavalent formulations, with and without Alhydrogel, were also tested in rats, in comparison with the corresponding tetravalent *Shigella* GMMA formulation with Alhydrogel, and the bivalent *Salmonella* formulation, with and without Alhydrogel. Rats were injected twice intramuscolarly (i.m.) 28 days apart, with each dose containing 0.6 µg of OAg *Shigella* GMMA and 1.0 µg of *Salmonella* glycoconjugate, corresponding to 1/25 of the highest expected human dose of each component.

**Figure 2:**
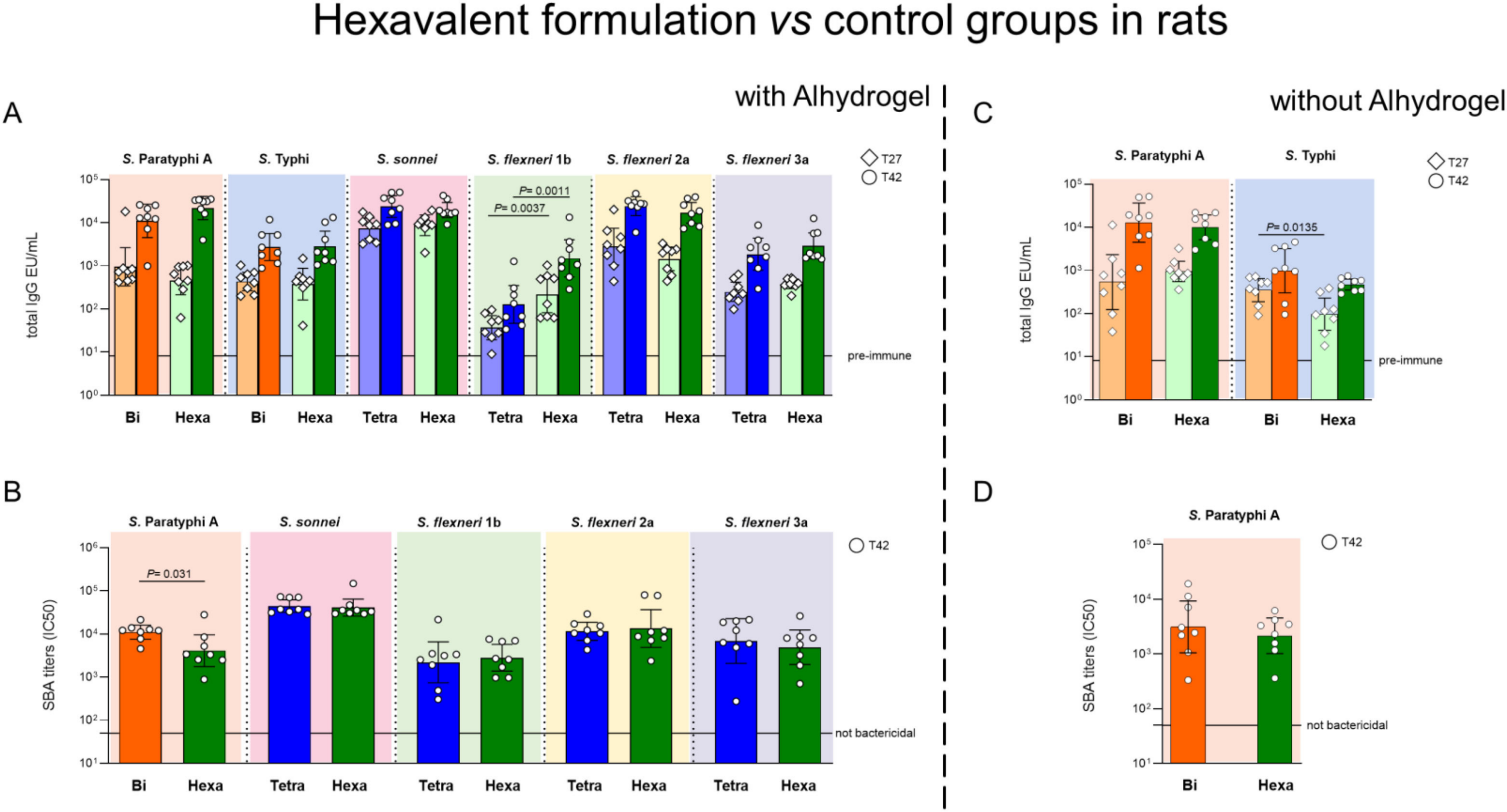
Hexavalent formulation (Hexa, green) tested in rats with and without Alhydrogel in comparison to bivalent *Salmonella* glycoconjugate (Bi, orange) and *Shigella* tetravalent GMMA (Tetra, blue). Sprague Dawley rats were immunized i.m. at day 0 and 28 with 0.6 µg/dose of each *Shigella* GMMA OAg and 1 µg/dose of each *Salmonella* glycoconjugate PS. Concentration of Alhydrogel, if present, was 0.7 mg/mL (Al^3+^). Sera collected at days 27 (T27) and 42 (T42) were analyzed by **(A, C)** ELISA for OAg-specific (*S. sonnei*, *S. flexneri* and *S.* Paratyphi A) or Vi total IgG (EU/mL). Sera collected at T42 were analyzed by **(B, D)** SBA for bactericidal titers expressed as IC50. Summary graphs of geometric mean units (bars) and individual levels (dots) are reported.

When comparing the hexavalent formulation with Alhydrogel to the corresponding bivalent formulation, both vaccines induced similar anti-Vi IgG responses after both injections. Although the hexavalent formulation elicited comparable anti-O:2 IgG response than the bivalent vaccine after the second injection, the bivalent formulation induced higher SBA titers (**Figures 2A, B**). No evidence of negative immuno-interference was observed in the responses against *Shigella* when comparing the hexavalent formulation to the tetravalent *Shigella* GMMA vaccine. Similar IgG responses were observed for most components—but for *S. flexneri* 1b, for which the hexavalent formulation induced higher IgG titers than the tetravalent vaccine, and comparable bactericidal titers were induced after the second injection by all formulations against all components (**Figures 2A, B**).

In the absence of Alhydrogel, no differences were observed in the IgG and SBA responses against *S.* Paratyphi A, while the hexavalent formulation elicited a lower anti-Vi IgG response after the first injection compared to the bivalent vaccine (**Figures 2B, D**).

The presence of Alhydrogel had a different impact on the specific IgG responses induced by the hexavalent formulation. Anti-Vi (both timepoints) and -*Shigella flexneri* 1b OAg (only post 1) IgG responses were higher when Alhydrogel was present in the formulation, whereas anti-*S. sonnei* (post 2) and *-S. flexneri* 2a (post 1) OAg IgG responses were stronger without it. The responses against *S. flexneri* 3a were similar regardless of Alhydrogel (**Supplementary Figure 2A**), and no differences were evidenced in SBA (**Supplementary Figure 2B**). After the second injection (T42), the IgG responses increased further for all components, with or without Alhydrogel.

### Immunogenicity study of the hexavalent formulation in rabbits

The hexavalent formulation with Alhydrogel was also tested in rabbits, and compared with the 4-valent *Shigella* GMMA (31, 36) and the bivalent *Salmonella* glycoconjugates (32) (Alfini R, submitted). Rabbits were injected i.m. at the maximum envisaged human dose of each antigen (15 µg *Shigella* OAg and 25 µg *Salmonella* PS).

**Figure 3:**
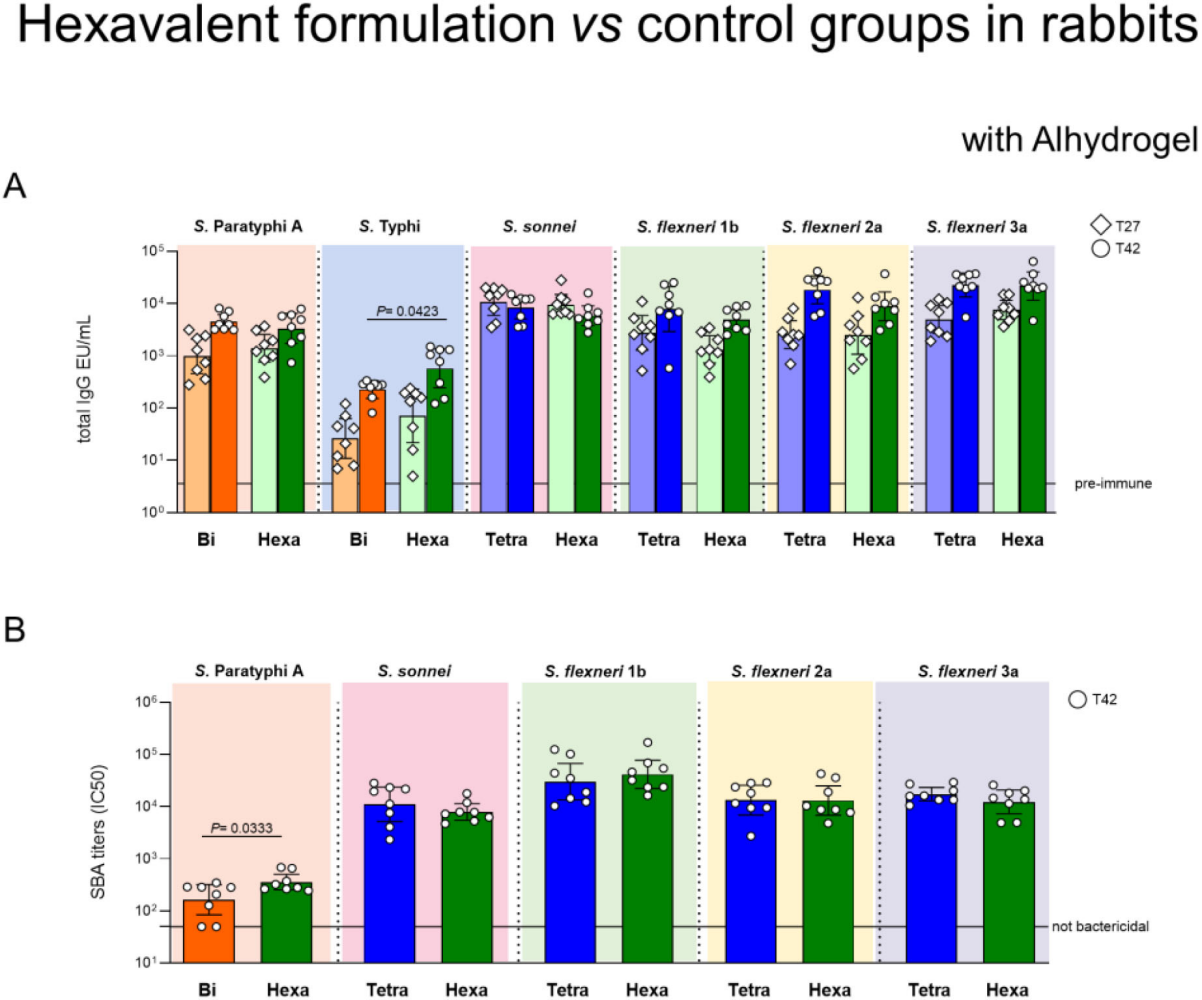
Hexavalent formulation (Hexa, green) tested in rabbits in comparison to bivalent *Salmonella* glycoconjugate (Bi, orange) and *Shigella* tetravalent GMMA (Tetra, blue). New Zealand rabbits were immunized intramuscolarly (i.m.) at day 0 and 28 with 15 µg/dose of each *Shigella* GMMA OAg and 25 µg/dose of each *Salmonella* glycoconjugate PS. Concentration of Alhydrogel was 0.7 mg/mL (Al^3+^). Sera collected at T27 and T42 were analyzed by **(A)** ELISA for OAg-specific (*S. sonnei*, *S. flexneri* and *S.* Paratyphi A) or Vi total IgG (EU/mL). Sera collected at T42 were analyzed by **(B)** SBA for bactericidal titers expressed as IC50. Summary graphs of geometric mean units (bars) and individual levels (dots) are reported. .

Similarly to mice and rats, no evidence of negative immuno-interference was observed in rabbits. Overall, the hexavalent formulation induced comparable responses to the *Salmonella* bivalent and *Shigella* 4-valent formulations (**Figures 3A, B**). Additionally, the presence of GMMA in the hexavalent formulation resulted in higher SBA response against *S.* Paratyphi A and higher IgG titers against *S.* Typhi post second immunization (**Figure 3B**). In rabbits, following the second injection, the IgG responses further increased.

### *S. sonnei* GMMA as carrier for *S*. Paratyphi A OAg

With the aim to simplify the hexavalent vaccine formulation, we explored the use of *S. sonnei* GMMA as carrier (35) for *S.* Paratyphi A O:2, potentially resulting in a single construct targeting the two different pathogens.

The 1-cyano-4-dimethylaminopyridine tetrafluoroborate (CDAP) chemistry (37) was selected for conjugation. Hydroxyl groups along the O:2 chain (**Supplementary Figure 3A**) were randomly activated using the CDAP cyanilating agent, followed by conjugation with GMMA, through formation of isourea linkages (**Supplementary Figure 4A**). Conjugate formation was confirmed by sodium dodecyl sulfate-polyacrylamide gel electrophoresis (SDS-page) and Western Blot (WB). In addition, the changes observed in the protein (SDS-page) and in the LPS (WB) patterns (**Supplementary Figures 4B, C**), indicated that, binding of O:2 involved both amino groups of LPS (**Supplementary Figure 3B**) and proteins of GMMA. High Performance Liquid Size Exclusion Chromatography (HPLC-SEC) analysis of the resulting conjugate was used to verify the absence of free O:2, while High Performance Anion Exchange Chromatography with Pulsed Amperometric Detection (HPAEC-PAD) allowed to confirm *S. sonnei* OAg content on GMMA and selectively assess O:2 density (38, 39). The final conjugate had an O:2 (*S.* Paratyphi A):OAg (*S. sonnei)*:protein w/w/w ratio of 0.4:0.3:1 (**Supplementary Table 1**).

### Immunogenicity study of *S. sonnei* GMMA-O:2 conjugate in mice

To begin, the GMMA conjugate was tested in mice, without Alhydrogel, in combination with the *S.* Typhi conjugate Vi-CRM_197_ and compared to a formulation containing *S. sonnei* GMMA physically mixed with the bivalent Vi-CRM_197_ + O:2-CRM_197_ glycoconjugates.

**Figure 4:**
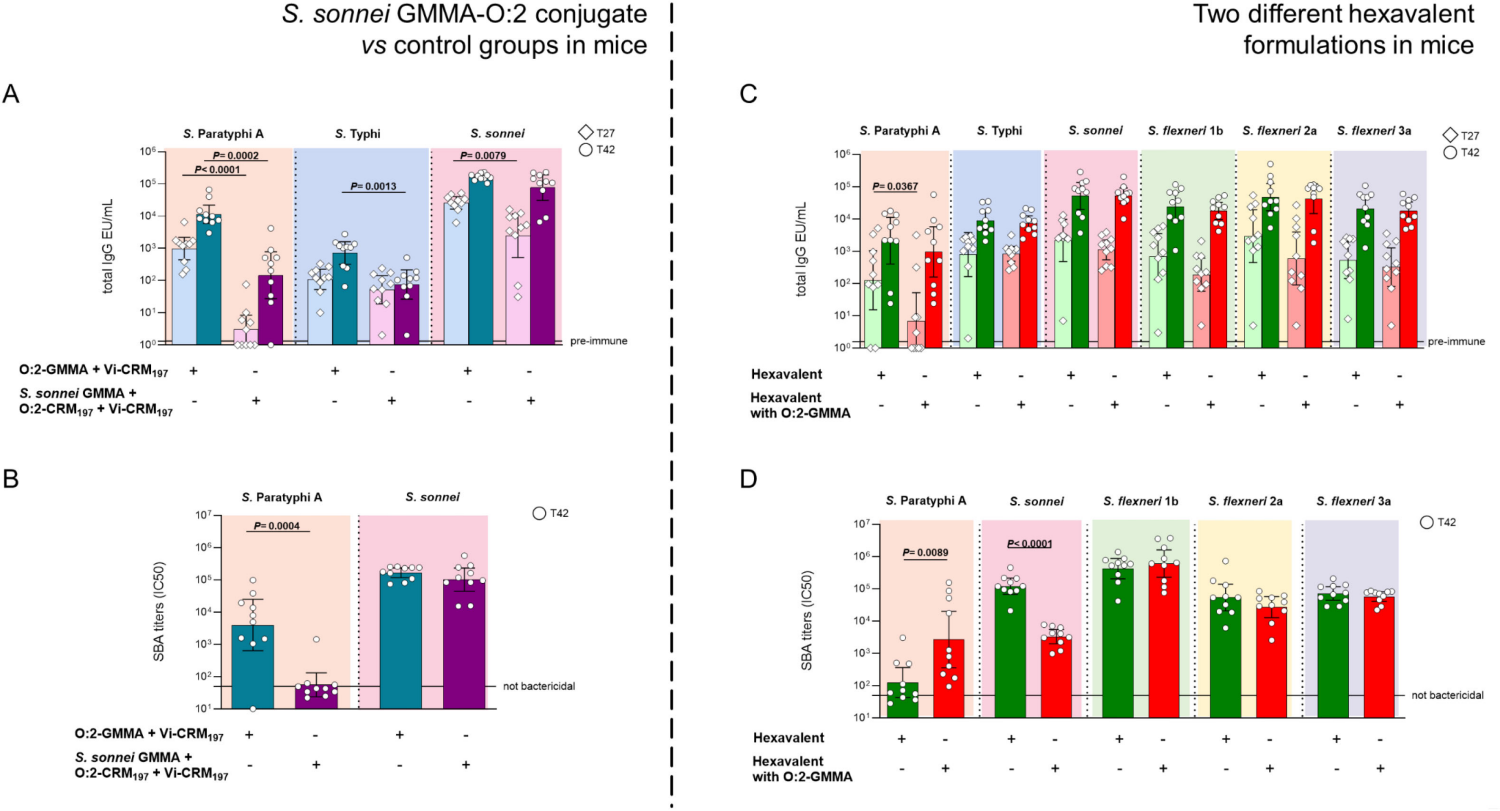
*S. sonnei* GMMA-O:2 conjugate + Vi-CRM_197_ compared to *S. sonnei* GMMA physically mixed with bivalent Vi-CRM_197_ + O:2-CRM_197_ in mice **(A,B)** and comparison of two different hexavalent formulations with O:2-CRM_197_ or O:2 linked to *S. sonnei* GMMA in mice **(C,D)**. CD1 mice were immunized i.p. at day 0 and 28 with **(A, B)** 0.5 µg of O:2, corresponding to 0.36 µg of *S. sonnei* OAg on GMMA and 0.5 µg of Vi, or **(C, D)** 0.15 µg OAg per each GMMA, 0.21 µg (O:2-GMMA) or 1 µg (O:2-CRM_197_) of O:2 and 1 µg of Vi. Alhydrogel at 0.7 mg/mL (Al^3+^) was only used in **(C, D)**. Sera collected at T27 and T42 were analyzed by **(A, C)** ELISA for OAg-specific (*S. sonnei*, *S. flexneri* and *S.* Paratyphi A) or Vi total IgG (EU/mL). Sera collected at T42 were analyzed by **(B, D)** SBA for bactericidal titers expressed as IC50. Summary graphs of geometric mean units (bars) and individual levels (dots) are reported.

The *S. sonnei* GMMA-O:2 conjugate elicited anti-O:2 IgG response significantly higher than the traditional O:2-CRM_197_ conjugate co-formulated with *S. sonnei* GMMA and Vi-CRM_197_ (**Figure 4A**). SBA titers mirrored the IgG response (**Figure 4B**). Additionally, anti-Vi IgG response was higher with O:2-GMMA, at day 42. The presence of O:2 on GMMA surface did not negatively impact neither anti-*S. sonnei* LPS IgG response, nor antibody functionality compared to unconjugated GMMA (**Figures 4A, B**).

Based on the encouraging results obtained, *S. sonnei* GMMA-O:2 construct was combined in a hexavalent formulation with Vi-CRM_197_ and the three other *S. flexneri* GMMA. The resulting formulation was tested in mice, in the presence of Alhydrogel, in comparison to the hexavalent formulation containing *S. sonnei* and *S. flexneri* 1b, 2a and 3a GMMA and the two *Salmonella* glycoconjugates.

A higher anti-O:2 IgG response was induced by O:2-CRM_197_ with respect to *S. sonnei* GMMA-O:2 conjugate after the first injection. However, the responses elicited by O:2 linked to GMMA resulted to be more bactericidal compared to those of O:2-CRM_197_ (**Figures 4C, D**), despite the lower dose of 0.21 µg O:2 on GMMA *vs* 1 µg of O:2 linked to CRM_197._ This difference was due to the fact that the formulations were prepared at the same *S. sonnei* OAg dose. A negative impact of the presence of O:2 linked on GMMA surface was observed on anti-*S. sonnei* responses in SBA after 2 injections (**Figures 4C, D**). No difference in anti-Vi or anti-*S. flexneri* IgG responses were observed between the two different formulations (**Figures 4C, D**).

## Discussion

*Shigella* is leading bacterial cause of diarrhea-related mortality and is associated with linear growth faltering and stunting (40). Its resistance to nearly all antimicrobial classes is increasing in prevalence and AMR isolates are becoming globally dominant, highlighting the urgent need of new interventions to prevent and treat *Shigella* infections (41). Tetravalent vaccine candidates are approaching phase 3 clinical trials (11), and the pathway for licensure of a *Shigella* vaccine has been defined (23). However, combination of a *Shigella* vaccine with other infant vaccine candidates could increase its commercial attractiveness and has recently been recommended (26).

Different criteria can drive the selection of an optimal combination vaccine, including diseases attributes (burden of disease, geography, target population, etc.), technical and clinical feasibility, stakeholder interest and speed of development. Licensed typhoid conjugate vaccines have been considered among the most easily and valuable vaccines to combine with a *Shigella* candidate (25). Moreover, a licensure pathway for combination of TCV with *S.* Paratyphi A is already in place (29).

Here, for the first time, we successfully combined a multivalent *Shigella* vaccine with a *Salmonella* bivalent formulation, containing both typhoid and paratyphoid conjugate vaccines, demonstrating that a combination of multiple GMMA and glycoconjugates is technically feasible and immunogenic. In addition, we showed that *Shigella* GMMA can be used as carrier for the *S.* Paratyphi A OAg, thus reducing the total number of components of the final formulation. We confirmed that in animal studies GMMA can significantly enhance the immune response elicited by conjugated PS, compared to traditional carrier protein (e.g. CRM_197_) (34, 35). However, in a hexavalent formulation, probably due to the presence of multiple GMMA, linkage of O:2 to one of the GMMA had a negative impact on the immune response elicited by the GMMA itself. That said, when choosing the components of a combination vaccine, it would be more desirable to start with candidates that are already advanced in clinical development, such as the 4-component *Shigella* GMMA vaccine, in phase 2 clinical trials in the target population (NCT05073003 and NCT06663436), and the bivalent glycoconjugate *Salmonella* vaccine, which has completed phase 1 (NCT05613205).

To account for the fact that the tetravalent *Shigella* vaccine contains Alhydrogel as adsorbent (31), we have identified formulation conditions to combine GMMA and *Salmonella* glycoconjugates, without impacting GMMA adsorption. We were also able to identify formulation conditions to maintain Vi-CRM_197_ not adsorbed as in licensed vaccines, and to modulate the percentage of adsorption of O:2-CRM_197_, that has been tested in the phase 1 clinical trial with and without adjuvant.

Recent studies suggest no need of Alhydrogel neither to increase GMMA magnitude, quality, or functionality of the antibody response induced (36, 42), nor to reduce their potential residual reactogenicity. *Shigella* GMMA formulated without Alhydrogel were well tolerated in rabbits (42) and analysis by a human monocyte activation test (MAT) showed that GMMA, with or without Alhydrogel, induce a similar level of IL-6 release (42, 43). Here, formulations with and without Alhydrogel were both tested in mice and rats. The rat model was selected as it most closely mimics human responses to *Shigella* (44). Moreover, this animal species is frequently used to evaluate the immunogenicity of multivalent vaccines in which immuno-interference among various antigens may occur (45, 46). In rats, no differences in functional antibody responses were detected irrespectively of the presence of Alhydrogel. In mice, instead, Alhydrogel increased immune response against all antigens, no difference was observed against *S. sonnei* only. The results in mice are different from what previously observed with GMMA only, in which presence of Alhydrogel did not result in an increased humoral response (36). We can argue that, in a more complex formulation, the presence of Alhydrogel could favor the response directed against each component in this animal model. Based on these results, it would be valuable to evaluate formulations with and without Alhydrogel in humans. If Alhydrogel is not required to enhance the immune response to the individual antigens, omission of the adjuvant could simplify manufacturing and analytical characterization of the final drug product. In general, combination vaccines may be less effective than vaccines administered separately, as one potential risk of multivalent vaccines failure could be negative immunological interference (47). Here, we have shown, with consistent results in all three different animal species tested, the possibility to combine the 6 different antigens with no major impact on the humoral immune response.

A combined vaccine against *Shigella* and *Salmonella* has the potential to address a huge medical need considering a burden of 148,202 deaths and 10.6 million disability-adjusted life-years (DALYs) caused by *Shigella;*110,029 deaths and 8.0 million DALYs caused by typhoid fever; 23,337 deaths and 1.6 million DALYs caused by paratyphoid fever, among all ages in 2019 (3). In addition, *Shigella* and TCV affect same regions in Africa and Asia, while *S.* Paratyphi A mainly affects Asia (3).

The *Shigella* and *Salmonella* vaccines share infants as target population. A possible obstacle to their combination could be related to the number of doses required and age of the target population. Indeed, TCV is a one-dose vaccine, while 2 doses could be needed for *Shigella* and/or *S*. Paratyphi A. Furthermore, to extend protection, recent data on persistency of antibodies elicited by TCV could suggest the need for a booster dose, ∼5 years after their primary dose, or delay in the vaccination (48).

Licensed TCVs are currently administered at the WHO 9-months or 15-months Expanded Programme on Immunization (EPI) time point. This age is compatible with the bivalent enteric fever vaccines in development, as paratyphoid A is uncommon in children aged <9 months (29). For *Shigella*, the peak of disease is in the second year of life and the current WHO Preferred Product Characteristics (PPC) preference is for a maximum of 2 primary doses in children <1 year of age (49). Development of such multicomponent vaccines also requires more thoughts about the clinical development pathway considering current regulatory guidelines (47, 50).

Despite some difficulties in selecting the optimal combination of a *Shigella* vaccine, this work provides the first evidence of the possibility of combining *Shigella* with *Salmonella* antigens and discusses the main criteria that could guide the development of multi-component vaccines, highlighting their benefits in addressing significant medical needs with a strong impact on global health.

## Materials and methods

### Preparation and characterization of *Shigella* GMMA

*Shigella* GMMA were produced from the following strains: *S. sonnei* 53G ΔtolR::kan ΔvirG::nadAB ΔmsbB2::cat ΔmsbB::erm, *S. flexneri* 1b Stansfield ΔtolR::frt ΔmsbB1a::frt ΔmsbB1b::frt, *S. flexneri* 2a 2457T ΔtolR::kan, ΔmsbB::cat, and *S. flexneri* 3a 6885 ΔtolR::kan, ΔmsbB::cat, and purified as previously described (31).

GMMA were purified and characterised as previously described (31). Total protein content was determined by micro Bicinchoninic acid assay (BCA) (Thermo Scientific, Waltham, MA, USA), total OAg amount by HPAEC-PAD and OAg to protein ratio was calculated. GMMA size was estimated by dynamic light scattering (dls), OAg molecular size was determined by HPLC-SEC after acetic acid extraction (51). Lipid A amount was quantified by Reversed Phase-High Performance Liquid Chromatography coupled with Mass Spectrometry (HPLC-RP MS) analysis (51).

### Preparation and characterization of *Salmonella* glycoconjugates

*S.* Paratyphy A OAg (O:2) was extracted from the ED199 ΔtolR strain as previously described (39). The Vi PS was purified from *Citrobacter freundii* NVGH 328 (52) and fragmented as reported in (53).

The O:2-CRM_197_ glycoconjugate was produced using the procedure reported in (37). Briefly, O:2 at 4.5 mg/mL in 0.5 M NaCl and 50 mM 1,4-diazabicyclo[2.2.2]octane (DABCO) pH 9 was activated adding a solution of 100 mg/mL CDAP in acetonitrile using a 0.2:1 weight ratio CDAP/OAg, corresponding to a final CDAP solution of 0.9 mg/mL. After 15 minutes in ice bath, CRM_197_ was added to the solution in a CRM_197_/OAg 1:1 weight ratio with final protein concentration of 3.8 mg/mL. The reaction was mixed for 2 h at room temperature (RT). At the end, 1M glycine at pH 9 was added (1:1 v/v with respect to the reaction mixture) to quench the reaction. The solution was gently mixed over night at RT.

The Vi-CRM_197_ glycoconjugate was synthesized as described in (53). Fragmented Vi was solubilized in 100 mM MES pH 6 at a concentration of 50 mg/mL. N-Hydroxysuccinimide (NHS) and then 1-Ethyl-3-(3-dimethylaminopropyl)carbodiimide (EDAC) were added to have 0.33 M NHS and EDAC/Vi repeating units molar ratio of 5. The reaction was mixed at room temperature (RT) for 1h and then the protein, previously derivatized with ADH (52), was added in a Vi: CRM_197_ w/w ratio of 1:1 to give a Vi concentration of 5.0 mg/mL in 20 mM MES pH 6 and mixed at RT for 2h.

The conjugates were purified by hydrophobic interaction chromatography (HIC) on a Phenyl HP column (Cytiva Life Sciences, Marlborough, MA, USA), loaded in 20 mM NaH_2_PO_4–_3 M NaCl at pH 7.2. The purified conjugate was eluted in 20 mM NaH_2_PO_4_ at pH 7.2 and the collected fractions were exchanged against Phosphate-buffered saline (PBS) by Amicon Ultra (Merck, Darmstadt, Germany) 30 kDa cut-off.

Purified conjugates were characterized by micro BCA (Thermo Scientific, Waltham, MA, USA) and HPAEC-PAD (38, 39) for total protein and total PS content, respectively, and the PS to protein ratio was calculated. Free O:2 was separated through reverse phase-solid phase extraction (SPE) using Vydac C4 SPE cartridges (32) and quantified by HPAEC-PAD, while for free Vi the unconjugated saccharide was estimated by HPAEC-PAD method (38) after conjugate coprecipitation with deoxycholate (39, 54). Conjugates formation was verified by HPLC-SEC, comparing the conjugates with unconjugated CRM_197_ (55).

### Preparation and characterization of *S. sonnei* GMMA-O:2 conjugate

CDAP chemistry was used for direct conjugation of O:2 to *S. sonnei* GMMA. The O:2, at 5 mg/mL final concentration, was solubilized in an ice bath at 0 °C in DABCO 50 mM at pH 9. CDAP 100 mg/mL solution in acetonitrile was added to give a final concentration of 2.5 mg/mL. After 15 min, GMMA were added to a final concentration of 2.8 mg/mL (protein-based) and the reaction mixture was maintained for 2 h at RT. After conjugation time, 1 M glycine in 50 mM sodium phosphate at pH 7 was added in equal volume with respect to the reaction mixture to quench eventual residual cyanoester groups, and the reaction mixture was kept at 2–8 °C overnight. The GMMA-O:2 conjugate was purified via diafiltration with Amicon centrifugal filters (Sigma, cut-off 300 kDa) against PBS, 10 diavolumes.

Purified GMMA conjugate was characterized for total protein content by micro BCA (Thermo Scientific, Waltham, MA, USA); total O:2 and *S. sonnei* OAg were quantified by HPAEC-PAD following distinct hydrolysis and chromatographic conditions (38, 39) and O:2 to protein ratio was calculated. GMMA size was estimated by DLS (51). Conjugate formation was verified by SDS-page/WB analyses, comparing the conjugate with unconjugated GMMA and O:2 + GMMA in physical mixture (56).

### Preparation and characterization of multivalent formulations

All the formulations without Alhydrogel were prepared by diluting antigens in NaCl 154 mM NaH_2_PO_4–_20 mM pH 6.5 or in PBS (for the study with *S. sonnei* GMMA-O:2 conjugate in mice).

Hexavalent vaccines formulations with Alhydrogel were prepared by firstly adsorbing, under continuous stirring, *S. sonnei* GMMA, or *S. sonnei* GMMA-O:2 conjugate, and *S. flexneri* 1b, 2a and 3a GMMA on Alhydrogel to reach the final concentrations of 120 μg/mL total OAg (30 μg/mL each OAg) and 0.7 mg/mL Al^3+^. After 2 hours, O:2-CRM_197_ (50 μg/mL OAg final concentration) was added and the suspension was mixed for 1 hour. Then, NaH_2_PO_4–_100 mM pH 6.5 (20 mM in the final formulation) and 1540 mM (154 mM in the final formulation) NaCl solution were added, followed by continuous stirring for 15 min. Finally, Vi-CRM_197_ (50 μg/mL Vi final concentration) was added, followed by continuous stirring for 15 h ± 4 h.

Further dilutions for immunogenicity studies were performed with Alhydrogel diluent (0.7 mg/mL Al^3+^ in NaCl 154 mM NaH_2_PO_4–_20 mM pH 6.5).

Four-component *Shigella* GMMA formulation with Alhydrogel was prepared as reported in (31).

Bivalent O:2-CRM_197_ + Vi-CRM_197_ with Alhydrogel formulation was prepared by firstly adsorbing, under continuous stirring, O:2-CRM_197_ (50 μg/mL OAg final concentration) on Alhydrogel (0.7 mg/mL Al^3+^ final concentration). After 2 hours, NaH_2_PO_4–_100 mM pH 6.5 and 1540 mM NaCl solution were added to reach, respectively, 20 mM and 154 mM in the final formulation, followed by continuous stirring for 30 min. Finally, Vi-CRM_197_ (50 μg/mL Vi final concentration) was added, followed by continuous stirring for 15 h ± 4 h.

Further dilutions for immunogenicity studies were performed with Alhydrogel diluent (0.7 mg/mL Al^3+^ in NaCl 154 mM NaH_2_PO_4–_20 mM pH 6.5).

All formulations with Alhydrogel were characterized in terms of not adsorbed antigens. Drug product supernatants collected after 0.2 µm centrifugal filtration by nanosep were analyzed by HPLC-RP MS (51) or HPAEC-PAD (38, 39) to quantify unabsorbed lipid A GMMA content and O:2/Vi content, respectively.

### *In vivo* studies

“GSK is committed to the Replacement, Reduction and Refinement of animal studies (3Rs). Non-animal models and alternative technologies are part of our strategy and employed where possible. When animals are required, application of robust study design principles and peer review minimises animal use, reduces harm and improves benefit in studies”. Animal studies were performed at the GSK Animal Facility (Siena, Italy), in compliance with the relevant guidelines (Italian D.Lgs. n. 26/14 and European directive 2010/63/UE) and the institutional policies of GSK. The animal protocols were approved by the Italian Ministry of Health (project No. 643/2021-PR, approval date 12/08/2021).

Number of animals per group was selected based on historical data variability and considering log normal distribution, in order to discriminate at least 4-fold difference between groups with 80% power and α of 0.05.

Female, 5 weeks old CD1 mice (10 per group) were vaccinated intraperitoneally (i.p.) with 200 µL of formulated antigens at study day 0 and 28. Individual sera were collected at days 27 and42.

Female, 8 weeks old, Sprague Dawley rats (8 per group) were vaccinated i.m. with 200 µL of formulated antigens at study day 0 and 28. Individual sera were collected at days 27 and 42.

Female New Zealand White rabbits Crl: KBL(NZW) (8 per group) were vaccinated intramuscularly (i.m.) with 500 µL of formulated antigens at study day 0 and 28. Single sera were collected on study days 27 and 42. Maximum volume of blood was sampled according to ethic’s recommendations.

### Sera analyses

Sera collected at different time points were analyzed by ELISA according to procedures already published (32, 36, 57), using as ELISA coating the following antigens: *S. sonnei* LPS at the concentration of 0.5 µg/mL in PBS, *S. flexneri* 1b OAg at the concentration of 2 µg/mL in Carbonate Buffer, *S. flexneri* 2a OAg at the concentration of 5 µg/mL in carbonate buffer, *S. flexneri* 3a OAg at the concentration of 1 µg/mL in PBS, *Salmonella* Paratyphi A OAg at the concentration of 2 µg/mL in carbonate buffer, Vi PS at the concentration of 1 µg/mL in phosphate buffer. Plates were blocked 1 h at 25 °C with PBS milk 5% and subsequently incubated for 2 h at 25 °C with the sera diluted 1:100, 1:4,000 and 1:160,000 in PBS-Tween 0.05% 0.1% BSA or PBS milk 5%. Bound antibodies were then detected after 1 h incubation at 25 °C with an enzyme-labeled secondary antibody (anti-mouse, anti-rat or anti-rabbit IgG-alkaline phosphatase) in PBS-Tween 0.05% 0.1% BSA.

The presence of immunoreacting anti-*S. sonnei*, *S. flexneri* 1b, 2a, 3a, *Salmonella* Paratyphi A OAg and Vi antibodies was detected after 1 h incubation at 25 °C with p-nitrophenyl phosphate substrate (Sigma-Aldrich) solution, through formation of a yellow color detected by absorbance at 405 nm subtracted by the absorbance at 490 nm. The samples were tested in comparison to calibrated mouse, rat or rabbit anti-antigens specific reference standard sera using a sigmoidal five parameter logistic (5 PL) interpolation of sample on the standard curve. Results were expressed in ELISA units/mL determined relative to the reference serum. One ELISA unit equals the reciprocal of the dilution of the reference serum that yields an Optical Density (OD) of 1 in the assay.

Individual serum samples collected at day 42 were also tested against bacterial strains (*S. sonnei* 53G virG::cat (58), *S. flexneri* 1b, Stansfield NTCT 5 strain; *S. flexneri* 2a, 2457T strain and *S. flexneri* 3a, NCTC9989 strain; *S.* Paratyphi A ED199 strain) in SBA based on luminescent readout as previously described (59–61). SBA methods used have been fully characterized for analysis of rabbit sera supporting toxicology studies and for analysis of human sera (60, 62, 63). Baby rabbit complement (BRC) was used 20% for *S. sonnei* and *S.* Paratyphi A, 15% in the case of *S. flexneri* 1b and 3a, and 7.5% for *S. flexneri* 2a. Phosphate-buffered saline (PBS) and LB medium (only in the case of *S. flexneri* 1b strain) were used for serum and bacteria dilutions for preparation of the reaction mix. Results of the assay were expressed as the IC50, the reciprocal serum dilution that resulted in a 50% reduction of luminescence and thus corresponding to 50% growth inhibition of the bacteria present in the assay. For data analysis, a 4-parameter non-linear regression was applied to raw luminescence data obtained at different dilutions tested for each serum sample. Fitting was performed by weighting the data for the inverse of luminescence^2. To no bactericidal sera, a titer equal to half of the first dilution of sera tested was assigned (i.e., (50)).

### Statistics

Statistical analysis was performed using GraphPad Prism 10 under the assumption of log−normality (64, 65), which was adopted to account for the biological variability typically observed in immunological measurements. For two−group comparisons, Welch’s t−tests assuming log−normal data distribution were performed; all P values and GMRs with 95% CIs are reported in **Supplementary Table 2**. Likewise, a paired t−test based on a log−normal data distribution was used to evaluate the responses induced by the same formulation at different timepoints.

## Data Availability

The original contributions presented in the study are included in the article/**Supplementary Material**, further inquiries can be directed to the corresponding author/s.
