# Supplementary material for: Preclinical evaluation of multivalent vaccine combinations against Shigella and Salmonella infections
Source: Front Immunol. 2026 Apr 22;17:1807547. doi: 10.3389/fimmu.2026.1807547 (PMC13143966; doi:10.3389/fimmu.2026.1807547)
Supplement: Supplementary file 1 [file SupplementaryFile1.docx]

**SUPPLEMENTARY INFORMATION**

**Preclinical evaluation of multivalent vaccine combinations against *Shigella* and *Salmonella* infections**

Roberta Di Benedetto^1,2^*, Rebecca Nappini^1^*, Salvatore Gemmellaro^1^, Federica Boretto^1^, Valentina Caradonna^1^, Martina Carducci^1^, Paola Cescutti^2^, Omar Rossi^1^, Francesca Mancini^1^, Francesco Berlanda Scorza^1^, Simona Rondini^1^, Carlo Giannelli^1^, Renzo Alfini^1^, Francesca Micoli^1#^

^1^ GSK Vaccines Institute for Global Health (GVGH), via Fiorentina 1, 53100 Siena, Italy

^2^ Department of Life Sciences, University of Trieste, via L. Giorgieri 1, 34127 Trieste, Italy

* Equally contributed to this work and share first authorship

^#^ Corresponding author, Francesca Micoli [francesca.x.micoli@gsk.com](mailto:francesca.x.micoli@gsk.com)

**Table S1.** Main characteristics of GMMA and glycoconjugates used to formulate the hexavalent vaccine.

| Sample | PS/protein weight ratio | Free PS % | Lipid A/OAg (nmol/µg) | Lipid A/total protein  (nmol/µg) | PS molecular size (MP)  distribution (KDa) | Particle Size  Z-average (diameter in nm)  PDI |
| --- | --- | --- | --- | --- | --- | --- |
| S. sonnei GMMA | 0.32 | na | 0.56 | 0.18 | 247.3  18.7  2.2 | 162.8  0.206 |
| S. flexneri 1b GMMA | 1.18 | na | 0.39 | 0.46 | 13.8  1.7 | 108.9  0.18 |
| S. flexneri 2a GMMA | 1.11 | na | 0.22 | 0.25 | 47.1  14.2  1.8 | 109.7  0.14 |
| S. flexneri 3a GMMA | 1.17 | na | 0.37 | 0.43 | 15.5  1.8 | 82.5  0.1 |
| Vi-CRM_197_ | 0.41 | 10.2 | na | na | 46.5 | na |
| O:2-CRM_197_ | 0.69 | 4.7 | na | na | 100 16 | na |
| O:2- *S. sonnei* GMMA | 0.42  (O:2/protein) 0.3  (*S. sonnei* OAg/protein) | <5 (O:2) | 0.5 (*S. sonnei* OAg) | 0.14 | na | 121.8 0.22 |

na: not applicable

**Table S2.** P values and geometric mean ratios (GMRs) with 95% CIs obtained using a log‑normal Welch’s t‑test to compare the immune responses between two groups.

**Immunogenicity study of the hexavalent formulation in mice**

| **Response** | **Timepoint** | **Hexavalent *vs*  control group* with Alum** | | | **Hexavalent *vs*  control group* without Alum** | | | **Hexavalent with Alum *vs*  without Alum** | | |
| --- | --- | --- | --- | --- | --- | --- | --- | --- | --- | --- |
|  |  | **P value** | **GMR** | **GMR 95% CI** | **P value** | **GMR** | **GMR 95% CI** | **P value** | **GMR** | **GMR 95% CI** |
| ***S.* Paratyphi A** | IgG day 27 | 0.3435 | 2.919 | 0.2886 to 29.51 | 0.174 | 1.752 | 0.7418 to 4.140 | 0.0164 | 0.08475 | 0.01225 to 0.5861 |
|  | IgG day 42 | 0.0465 | 12.82 | 1.047 to 157.0 | 0.0004 | 36.6 | 7.132 to 187.9 | 0.0011 | 0.04121 | 0.007554 to 0.2248 |
|  | SBA day 42 | 0.0149 | 7.244 | 1.563 to 33.56 | N/A | N/A | N/A | <0.0001 | 0.02297 | 0.009921 to 0.05321 |
| ***S.* Typhi** | IgG day 27 | 0.3492 | 1.986 | 0.4256 to 9.263 | 0.0015 | 3.946 | 1.821 to 8.548 | <0.0001 | 0.1165 | 0.05502 to 0.2469 |
|  | IgG day 42 | 0.2363 | 2.411 | 0.5212 to 11.15 | 0.007 | 4.401 | 1.600 to 12.10 | <0.0001 | 0.05156 | 0.02239 to 0.1187 |
| ***S. sonnei*** | IgG day 27 | 0.3393 | 0.5729 | 0.1736 to 1.891 | N/A | N/A | N/A | 0.6284 | 1.298 | 0.4262 to 3.954 |
|  | IgG day 42 | 0.6252 | 1.907 | 0.1245 to 29.23 | N/A | N/A | N/A | 0.2595 | 4.277 | 0.3102 to 58.96 |
|  | SBA day 42 | 0.5758 | 0.8788 | 0.5439 to 1.420 | N/A | N/A | N/A | 0.8779 | 0.9624 | 0.5737 to 1.614 |
| ***S. flexneri* 1b** | IgG day 27 | 0.1965 | 1.501 | 0.7930 to 2.840 | N/A | N/A | N/A | <0.0001 | 0.1445 | 0.06703 to 0.3115 |
|  | IgG day 42 | 0.0436 | 3.944 | 1.047 to 14.85 | N/A | N/A | N/A | 0.0019 | 0.2866 | 0.1393 to 0.5897 |
|  | SBA day 42 | 0.8619 | 1.058 | 0.5400 to 2.073 | N/A | N/A | N/A | 0.0038 | 0.3086 | 0.1472 to 0.6473 |
| ***S. flexneri* 2a** | IgG day 27 | 0.8338 | 1.111 | 0.3907 to 3.162 | N/A | N/A | N/A | 0.1853 | 0.5487 | 0.2197 to 1.371 |
|  | IgG day 42 | 0.36 | 0.7266 | 0.3546 to 1.489 | N/A | N/A | N/A | 0.3746 | 0.7347 | 0.3595 to 1.502 |
|  | SBA day 42 | 0.7734 | 0.9153 | 0.4831 to 1.734 | N/A | N/A | N/A | 0.0001 | 0.3293 | 0.2141 to 0.5066 |
| ***S. flexneri* 3a** | IgG day 27 | 0.262 | 1.589 | 0.6845 to 3.689 | N/A | N/A | N/A | 0.012 | 0.2326 | 0.07790 to 0.6947 |
|  | IgG day 42 | 0.759 | 1.102 | 0.5703 to 2.130 | N/A | N/A | N/A | 0.009 | 0.2954 | 0.1234 to 0.7071 |
|  | SBA day 42 | 0.006 | 0.3603 | 0.1809 to 0.7174 | N/A | N/A | N/A | 0.0002 | 0.2245 | 0.1129 to 0.4464 |

*For *S.* Paratyphi A and *S.* Typhi responses, control group is the bivalent formulation; for *S. sonnei* and *S.* *flexneri* 1b, 2a and 3a responses, control group is the tetravalent formulation.

**Immunogenicity study of the hexavalent formulation in rats**

| **Response** | Timepoint | **Hexavalent *vs*  control group* with Alum** | | | **Hexavalent *vs*  control group* without Alum** | | | **Hexavalent with Alum *vs*  without Alum** | | |
| --- | --- | --- | --- | --- | --- | --- | --- | --- | --- | --- |
|  |  | **P value** | **GMR** | **GMR 95% CI** | **P value** | **GMR** | **GMR 95% CI** | **P value** | **GMR** | **GMR 95% CI** |
| ***S.* Paratyphi A** | IgG day 27 | 0.2068 | 0.4859 | 0.1503 to 1.571 | 0.4092 | 1.772 | 0.3966 to 7.914 | 0.0955 | 2.058 | 0.8638 to 4.903 |
|  | IgG day 42 | 0.1594 | 1.986 | 0.7344 to 5.370 | 0.6475 | 0.7823 | 0.2495 to 2.452 | 0.0613 | 0.4589 | 0.2019 to 1.043 |
|  | SBA day 42 | 0.031 | 0.3715 | 0.1543 to 0.8948 | 0.5182 | 0.6892 | 0.2045 to 2.323 | 0.1982 | 0.5238 | 0.1875 to 1.464 |
| ***S.* Typhi** | IgG day 27 | 0.7316 | 0.8645 | 0.3483 to 2.145 | 0.0135 | 0.2781 | 0.1057 to 0.7319 | 0.0187 | 0.2583 | 0.08668 to 0.7696 |
|  | IgG day 42 | 0.9323 | 1.041 | 0.3875 to 2.794 | 0.1901 | 0.4769 | 0.1445 to 1.574 | 0.0009 | 0.1654 | 0.07239 to 0.3780 |
| ***S. sonnei*** | IgG day 27 | 0.6448 | 1.167 | 0.5780 to 2.355 | N/A | N/A | N/A | 0.1504 | 1.509 | 0.8396 to 2.713 |
|  | IgG day 42 | 0.5346 | 0.8264 | 0.4323 to 1.579 | N/A | N/A | N/A | 0.0123 | 1.874 | 1.174 to 2.992 |
|  | SBA day 42 | 0.7361 | 0.9211 | 0.5495 to 1.544 | N/A | N/A | N/A | 0.3205 | 0.7861 | 0.4744 to 1.303 |
| ***S. flexneri* 1b** | IgG day 27 | 0.0037 | 5.922 | 2.013 to 17.42 | N/A | N/A | N/A | 0.0387 | 0.2757 | 0.08208 to 0.9258 |
|  | IgG day 42 | 0.0011 | 11.71 | 3.241 to 42.30 | N/A | N/A | N/A | 0.0699 | 0.3086 | 0.08537 to 1.115 |
|  | SBA day 42 | 0.6714 | 1.271 | 0.3821 to 4.230 | N/A | N/A | N/A | 0.381 | 1.622 | 0.5107 to 5.149 |
| ***S. flexneri* 2a** | IgG day 27 | 0.2084 | 0.5196 | 0.1777 to 1.520 | N/A | N/A | N/A | 0.0044 | 3.207 | 1.538 to 6.685 |
|  | IgG day 42 | 0.2801 | 0.7008 | 0.3554 to 1.382 | N/A | N/A | N/A | 0.6131 | 0.8534 | 0.4418 to 1.649 |
|  | SBA day 42 | 0.7645 | 1.157 | 0.4030 to 3.321 | N/A | N/A | N/A | 0.5938 | 0.7313 | 0.2136 to 2.504 |
| ***S. flexneri* 3a** | IgG day 27 | 0.1066 | 1.539 | 0.8959 to 2.642 | N/A | N/A | N/A | 0.1587 | 1.72 | 0.7718 to 3.833 |
|  | IgG day 42 | 0.3506 | 1.566 | 0.5766 to 4.254 | N/A | N/A | N/A | 0.3984 | 0.663 | 0.2401 to 1.831 |
|  | SBA day 42 | 0.6164 | 0.7208 | 0.1820 to 2.855 | N/A | N/A | N/A | 0.9592 | 0.9686 | 0.2596 to 3.614 |

*For *S.* Paratyphi A and *S.* Typhi responses, control group is the bivalent formulation; for *S. sonnei* and *S.* *flexneri* 1b, 2a and 3a responses, control group is the tetravalent formulation.

**Immunogenicity study of the hexavalent formulation in rabbits**

| **Response** | Timepoint | **Hexavalent *vs*  control group* with Alum** | | |
| --- | --- | --- | --- | --- |
|  |  | **P value** | **GMR** | **GMR 95% CI** |
| ***S.* Paratyphi A** | IgG day 27 | 0.4062 | 1.427 | 0.5839 to 3.489 |
|  | IgG day 42 | 0.3443 | 0.7352 | 0.3686 to 1.466 |
|  | SBA day 42 | 0.0333 | 2.17 | 1.077 to 4.374 |
| ***S.* Typhi** | IgG day 27 | 0.1327 | 2.712 | 0.7072 to 10.40 |
|  | IgG day 42 | 0.0423 | 2.533 | 1.040 to 6.165 |
| ***S. sonnei*** | IgG day 27 | 0.7257 | 0.8947 | 0.4571 to 1.751 |
|  | IgG day 42 | 0.2333 | 0.7076 | 0.3899 to 1.284 |
|  | SBA day 42 | 0.3593 | 0.7099 | 0.3208 to 1.571 |
| ***S. flexneri* 1b** | IgG day 27 | 0.1013 | 0.4847 | 0.1997 to 1.176 |
|  | IgG day 42 | 0.3445 | 0.6358 | 0.2289 to 1.766 |
|  | SBA day 42 | 0.4665 | 1.379 | 0.5470 to 3.478 |
| ***S. flexneri* 2a** | IgG day 27 | 0.9734 | 0.9851 | 0.3788 to 2.562 |
|  | IgG day 42 | 0.0718 | 0.4834 | 0.2171 to 1.076 |
|  | SBA day 42 | 0.9602 | 0.9803 | 0.4235 to 2.269 |
| ***S. flexneri* 3a** | IgG day 27 | 0.2074 | 1.519 | 0.7680 to 3.004 |
|  | IgG day 42 | 0.8957 | 0.955 | 0.4555 to 2.002 |
|  | SBA day 42 | 0.2064 | 0.7118 | 0.4079 to 1.242 |

**For *S.* Paratyphi A and *S.* Typhi responses, control group is the bivalent formulation;

for *S. sonnei* and *S.* *flexneri* 1b, 2a and 3a responses, control group is the tetravalent formulation.

**Immunogenicity study of *S. sonnei* GMMA-O:2 conjugate in mice**

| **Response** | Timepoint | **[O:2-GMMA + ViCRM_197_] *vs*  [O:2-CRM_197_ + ViCRM_197_ + *S. sonnei* GMMA]** | | | **Hexavalent *vs*  hexavalent with O:2-GMMA** | | |
| --- | --- | --- | --- | --- | --- | --- | --- |
|  |  | **P value** | **GMR** | **GMR 95% CI** | **P value** | **GMR** | **GMR 95% CI** |
| ***S.* Paratyphi A** | IgG day 27 | <0.0001 | 0.003086 | 0.0009163 to 0.01039 | 0.0367 | 0.05428 | 0.003605 to 0.8172 |
|  | IgG day 42 | 0.0002 | 0.01269 | 0.002234 to 0.07208 | 0.4679 | 0.4483 | 0.04614 to 4.355 |
|  | SBA day 42 | 0.0004 | 0.01406 | 0.002024 to 0.09764 | 0.0089 | 21.58 | 2.467 to 188.8 |
| ***S.* Typhi** | IgG day 27 | 0.1957 | 0.4762 | 0.1487 to 1.525 | 0.9433 | 1.055 | 0.2102 to 5.293 |
|  | IgG day 42 | 0.0013 | 0.1044 | 0.03041 to 0.3583 | 0.645 | 0.8358 | 0.3726 to 1.875 |
| ***S. sonnei*** | IgG day 27 | 0.0079 | 0.09529 | 0.01943 to 0.4673 | 0.3763 | 0.5097 | 0.1038 to 2.504 |
|  | IgG day 42 | 0.0943 | 0.4667 | 0.1860 to 1.171 | 0.9845 | 1.01 | 0.3512 to 2.903 |
|  | SBA day 42 | 0.2411 | 0.6129 | 0.2582 to 1.455 | <0.0001 | 0.02706 | 0.01327 to 0.05520 |
| ***S. flexneri* 1b** | IgG day 27 | N/A | N/A | N/A | 0.1653 | 0.2725 | 0.04099 to 1.812 |
|  | IgG day 42 | N/A | N/A | N/A | 0.5782 | 0.7405 | 0.2398 to 2.287 |
|  | SBA day 42 | N/A | N/A | N/A | 0.5053 | 1.438 | 0.4655 to 4.444 |
| ***S. flexneri* 2a** | IgG day 27 | N/A | N/A | N/A | 0.1938 | 0.2034 | 0.01707 to 2.425 |
|  | IgG day 42 | N/A | N/A | N/A | 0.8618 | 0.8938 | 0.2347 to 3.403 |
|  | SBA day 42 | N/A | N/A | N/A | 0.2217 | 0.5054 | 0.1625 to 1.572 |
| ***S. flexneri* 3a** | IgG day 27 | N/A | N/A | N/A | 0.5473 | 0.5961 | 0.1013 to 3.507 |
|  | IgG day 42 | N/A | N/A | N/A | 0.766 | 0.8563 | 0.2884 to 2.542 |
|  | SBA day 42 | N/A | N/A | N/A | 0.4174 | 0.8043 | 0.4623 to 1.400 |

**
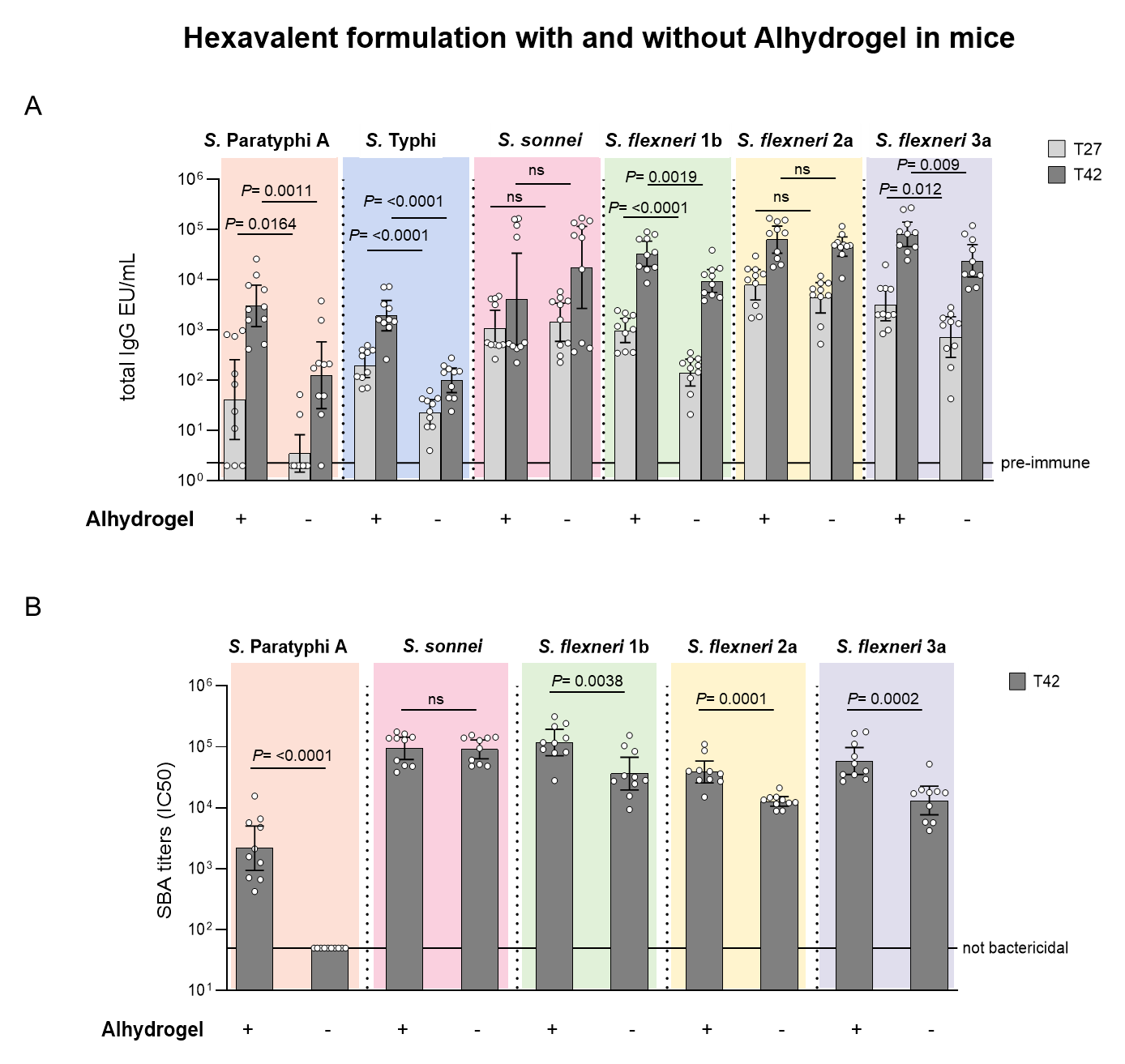
**

**Fig. S1.** Hexavalent formulation tested in mice with and without Alhydrogel. CD1 mice were immunized intraperitoneally (i.p.) at day 0 and 28 with 75 ng/dose of each *Shigella* GMMA OAg and 125 ng/dose of each *Salmonella* glycoconjugate PS. Concentration of Alhydrogel, if present, was 0.7 mg/mL (Al^3+^). Sera collected at days 27 (T27) and 42 (T42) were analysed by (A) ELISA for OAg-specific total IgG (*S. sonnei*, *S. flexneri* and *S.* Paratyphi A), or for Vi total IgG expressed as EU/mL. Sera collected at T42 were analysed by (B) SBA for species/serotype-specific bactericidal titers expressed as IC50. Summary graphs of geometric mean units (bars) and individual levels (dots) are reported.

.
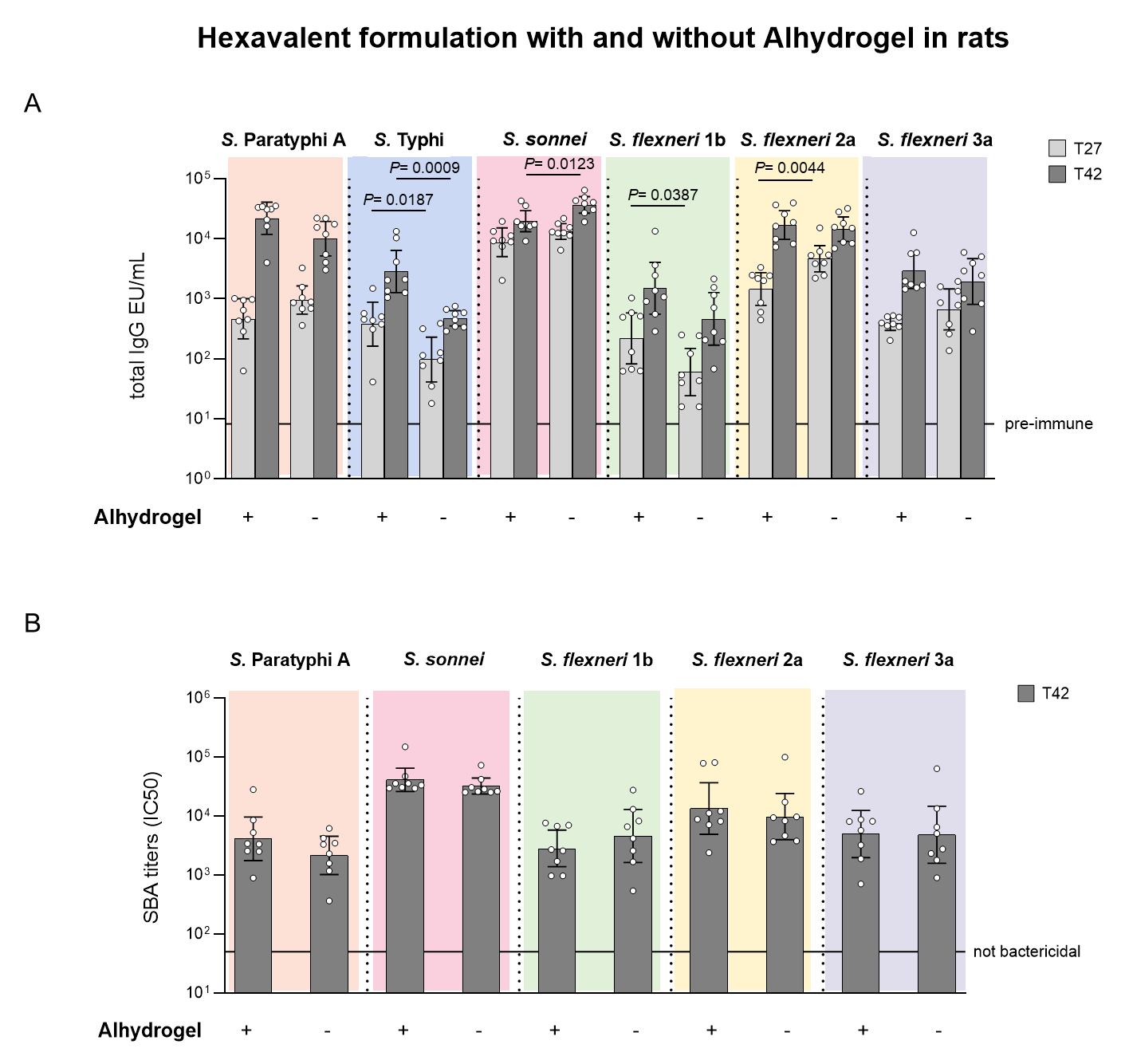


**Fig. S2.** Hexavalent formulation tested in rats with and without Alhydrogel. Sprague Dawley rats were immunized i.m. at day 0 and 28 with 0.6 µg/dose of each *Shigella* GMMA OAg and 1 µg/dose of each *Salmonella* glycoconjugate PS. Concentration of Alhydrogel, if present, was 0.7 mg/mL (Al^3+^). Sera collected at days 27 (T27) and 42 (T42) were analysed by (A) ELISA for OAg-specific total IgG (*S. sonnei*, *S. flexneri* and *S.* Paratyphi A), or for Vi total IgG expressed as EU/mL. Sera collected at T42 were analysed by (B) SBA for species/serotype-specific bactericidal titers expressed as IC50. Summary graphs of geometric mean units (bars) and individual levels (dots) are reported.

**
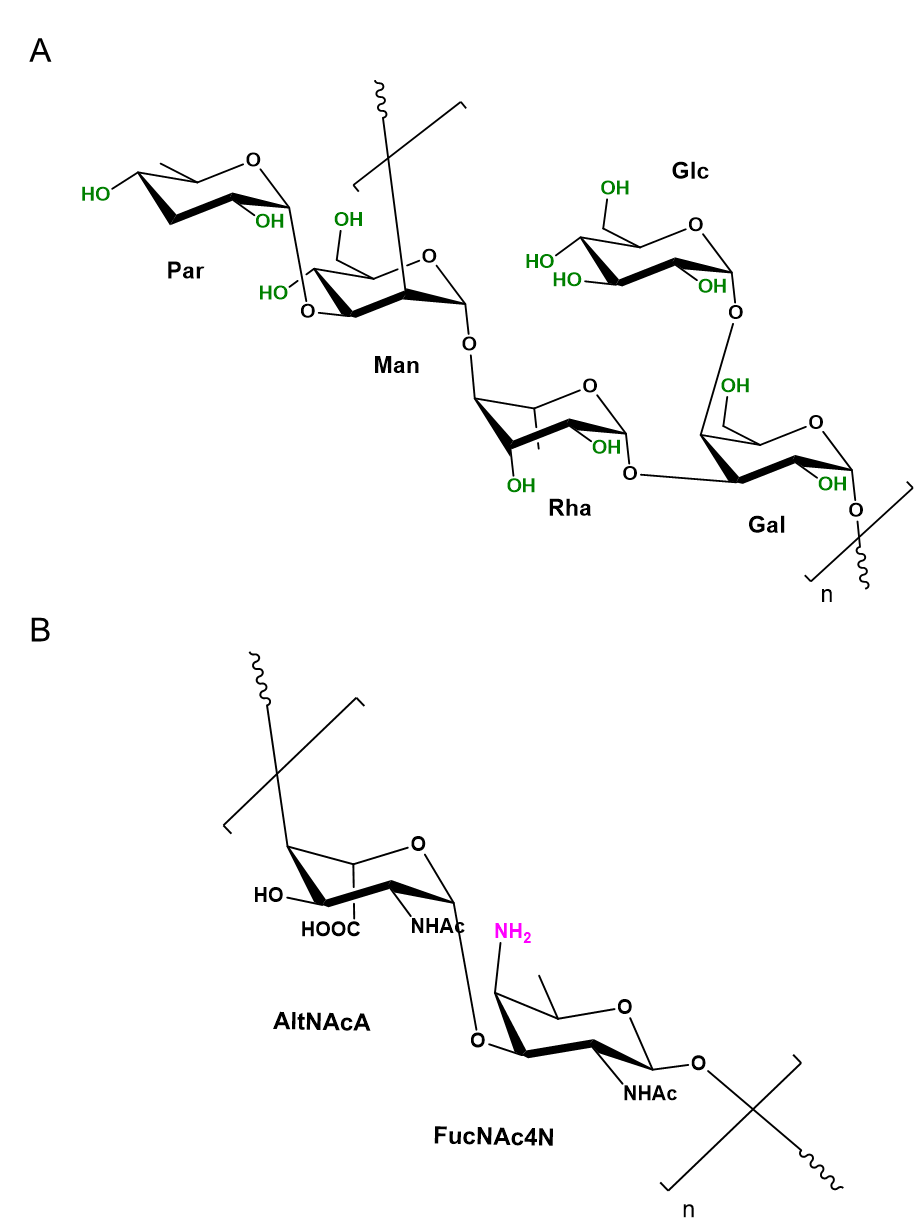
**

**Fig. S3.** (A) Hydroxyl groups in O:2 susceptible to activation with CDAP, highlighted in green; (B) amino groups in *S. sonnei* LPS OAg susceptible to conjugation with activated O:2, highlighted in pink.


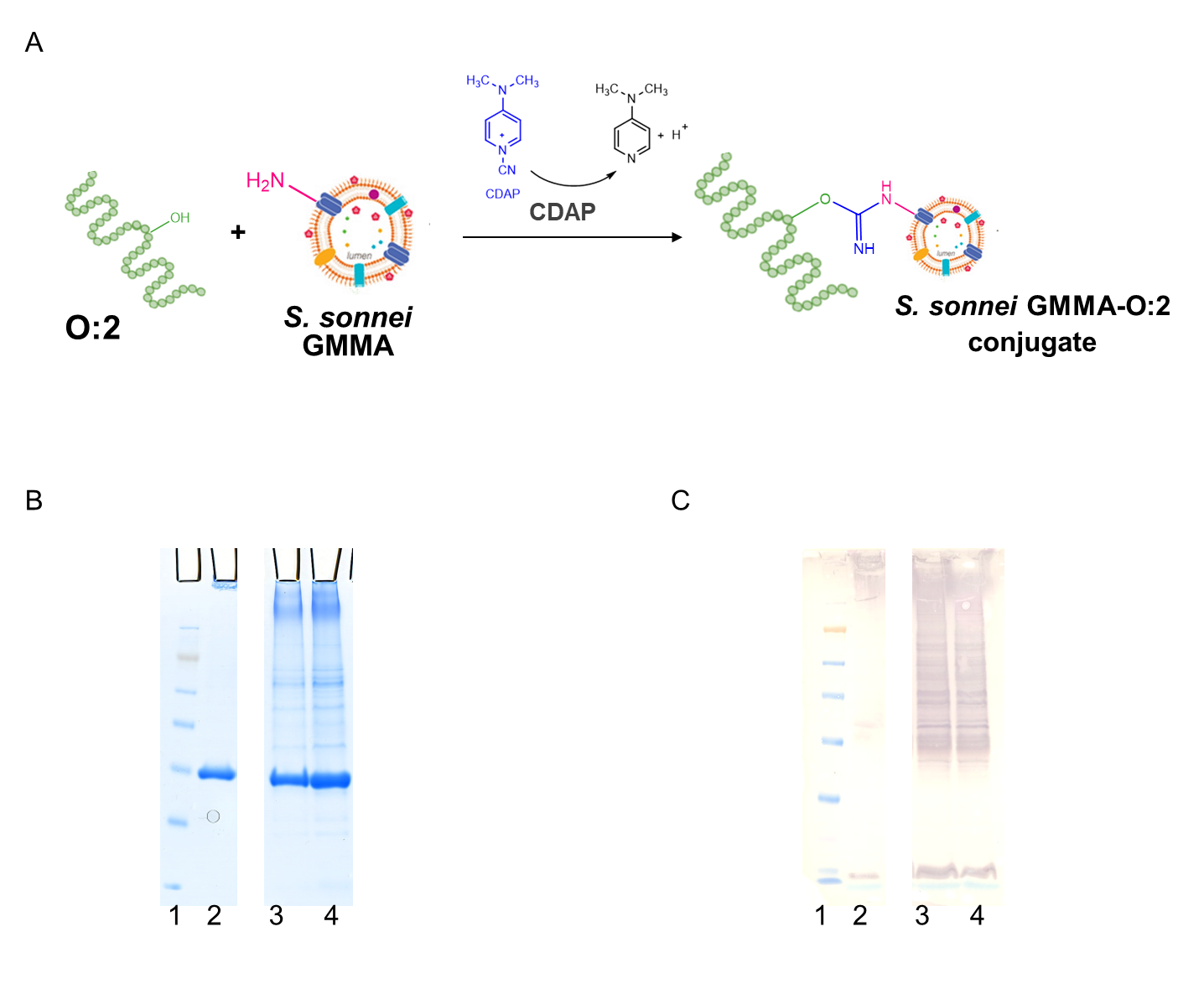


**Fig. S4.** (A) *S.* Paratyphi A O:2 was conjugated to *S. sonnei* GMMA through random activation of hydroxyl groups along O:2 chain using the cyanilating agent CDAP, followed by linkage to -NH_2_ groups on proteins and LPS of GMMA. (B) SDS-page and (C) anti-*S. sonnei* LPS WB analyses of the purified O:2-GMMA conjugate in comparison to corresponding unconjugated GMMA and O:2 + GMMA in physical mixture. Fifteen µg of conjugate and GMMA (protein based) and 15 µg of O:2 were loaded per well. Lane 1: marker, lane 2: O:2-GMMA conjugate, lane 3: O:2 + GMMA physical mixture, lane 4: GMMA.
